# Development and initial validation of the ILD-Anxiety-Questionnaire (IAQ): A new instrument for assessing disease specific fears in interstitial lung disease

**DOI:** 10.1177/14799731241274785

**Published:** 2024-08-26

**Authors:** Nikola M. Stenzel, Nina Piel, Klaus Kenn, Michael Kreuter

**Affiliations:** 1 530999 Psychologische Hochschule Berlin (PHB), Berlin, Germany; 2 9377 Philipps-Universität Marburg, Marburg, Germany; 3 MK: Mainz Center for Pulmonary Medicine, Departments of Pneumology, Mainz University Medical Center and of Pulmonary, Critical Care & Sleep Medicine, Marienhaus Clinic Mainz, Mainz, Germany

**Keywords:** Interstitial lung disease, idiopathic pulmonary fibrosis, disease-related fears, disease-specific fears, ILD-anxiety-questionnaire, IAQ, fear of progression, quality of life

## Abstract

Introduction: Multiple studies focusing on chronic lung diseases (i.e. COPD), have indicated that the quality of life (QoL) can be impacted by disease-related fears. In the context of Interstitial Lung Diseases (ILD), however, these have never been systematically examined. Therefore, the aim of the present study was to develop and evaluate an appropriate measuring tool, and to investigate the influence of disease-related anxieties on QoL in ILD. Method: *N* = 166 ILD patients participated in the study and completed an itempool on disease-related fears, based on the COPD-Anxiety-Questionnaire (CAF-R) and expert assessments. Further, demographic and psychological variables were assessed (anxiety: GAD-7, QoL: K-BILD; Beliefs about Health: KKG). Psychometric properties were analyzed (factor structure, reliability, validity). Regression analyses were used to calculate the differential predictive power of disease-related anxieties on QoL. Results: The factor structure was confirmed (Scales: Fear-of-Dependence-and-Progression, Fear-of-Social-Exclusion-and-Isolation, Fear-of-Physical-Activity, Fear-of-Dyspnea, and Sleep-related- Complaints). The Scales showed satisfying reliabilities (α = 0.68 to 0.89) and good validity. Disease-related anxieties proved to be differential predictors for different scales of the K-BILD (ß = −0.15 to ß = −0.58, all ps < .01). Conclusions: The ILD-Anxiety-Questionnaire (IAQ) is an easy-to-use, valid measurement tool for assessing disease-related anxieties. These vary in their impact on different aspects of QoL. Therefore, it might aid in specifying the indication for potential psychological supplementary interventions. Additional long-term studies are required to investigate how specific anxieties affect both overall and condition-specific QoL in diverse situations.

## Introduction

Interstitial lung disease (ILD) is an umbrella term for multiple conditions defined by diffuse inflammation and/or fibrosis of the pulmonary interstitium. ILDs can be divided into 4 main groups and the most prevalent forms include Idiopathic Pulmonary Fibrosis (IPF), Hypersensitivity Pneumonitis, Idiopathic nonspecific interstitial pneumonia (NSIP), systemic disease associated ILDs and Sarcoidosis.^1^ ILDs, especially fibrosing forms are associated with a significant symptom burden and a detrimental prognosis with a median survival of 3-5 years for IPF.^1^

Frequent symptoms in ILD are chronic dyspnea on exertion, cough, fatigue and reduced physical functioning in daily activities. In addition to a reduced quality of life (QoL), patients often suffer from accompanying psychological symptoms. Prevalence estimates for comorbid anxiety show wide ranges (21%-60%), depending on which screening tools are used.^2–6^ A recent study differentiated between clinical diagnosis and subclinical symptoms, with a reported prevalence of 33% for subclinical symptoms and 16% for clinically relevant anxiety symptoms.^7^ More severe anxiety symptoms were associated with poorer health, more severe dyspnea, but showed no or weak correlations with pulmonary function. Longer disease duration was associated with greater anxiety in IPF-patients.^8,9^

In our exploration of psychological assessments for ILD patients, we identify significant limitations with general measures such as the HADS (Hospital Anxiety and Depression Scale^10^). These tools are developed to assess psychological comorbidities in people with physical illnesses. However, they do not adequately consider the overlapping somatic symptoms that are experienced as part of living with ILD (e.g., HADS – “I feel slowed down”). An assessment instrument, *specifically designed* to measure disease specific quality of life in *ILD patients* is the King’s Brief Interstitial Lung Disease (K-BILD) Health Status Scale.^11,12^ It`s psychological scale includes concerns about lung problems in general, their intensification, and their impact on mood. However, it does not sufficiently capture more specific anxieties and worries (i.e. fear of dyspnea or physiological activity, or fear of social isolation). Therefore, we believe it lacks the detail needed to assess the full spectrum of psychological anxieties faced by ILD patients.

One concept that is receiving increasing attention in the context of chronic lung diseases is *disease-related fears*. This refers to fears that relate to the symptoms of the disease itself (e.g. dyspnea) or its progression.^13^ In the context of a chronically progressive illness, an increased preoccupation with illness-related topics can initially be seen as part of a ‘functional coping process’. However, if these fears increase, they may themselves become ‘dysfunctional’ and an additional burden for patients.^14^ Several studies in the field of other chronic lung diseases (e.g. COPD) have highlighted that disease-related fears can play a major role for health-related quality of life (HQoL) and illness-behavior. For example, Fear-of-Dyspnea and Fear-of-Physical-Activity may be associated with avoidance tendencies with regard to physical activity.^14–16^ Consequently, studies show that the extent of disease-related fears can predict the outcome of pulmonary rehabilitation.^16,17^ The results of these studies suggest that it may be important to address disease-related anxieties as part of treatment.^18^

However, the concept of disease-related fears has not yet been systematically investigated in ILD. Furthermore, the absence of a dedicated measurement instrument for evaluating this aspect poses a challenge. In contrast, other chronic respiratory conditions benefit from well-established measurement tools like the COPD-Anxiety-Questionnaire (CAF-R^14^), which is accessible in multiple languages, enabling the systematic assessment of disease-specific anxiety.

The aim of the present study was to systematically investigate disease-related anxiety in ILD for the first time. Consistently, the aim was to develop and evaluate a questionnaire that can systematically, reliably, and validly assess disease-related fears in ILD. Moreover, we aimed to investigate the impact of disease-related fears on HRQoL in ILD providing insights that could inform differential interventions in the long term.

## Methods

The construction and psychometric testing of the IAQ involved a multi-stage process: (a) development of the initial IAQ item pool, (b) construction of the final questionnaire through item, reliability and factor analyses, (c) assessment of the construct validity of the final questionnaire, (d) providing of normative data. This process is described in excerpts below. Details can be found in an additional document (Supplemental material S1, S2, S3).

### Development of the initial IAQ item pool

The development of the ILD-Anxiety-Questionnaire (IAQ) was based on the COPD-Anxiety-Questionnaire (CAF^19^), a self-assessment tool for evaluating disease-related fears in COPD patients (28 items, five-point Likert scale). Initially, CAF items were adapted to form the basis of the IAQ item pool. Two ILD experts with over 15 years of clinical and research experience reviewed the items, suggesting the removal of one specific to COPD and retaining the rest. Patient representatives were then consulted to identify any potential omissions based on their experiences. They highlighted the need for additional items addressing the limited public awareness of ILD. Suggestions were discussed and refined, resulting in two new items about the lack of understanding from others due to the disease’s obscurity. Finally, the initial IAQ item pool consisted of 29 items after eliminating redundancies and inconsistencies (Supplemental material S1 and S2).

### Data collection and study design

Patients were recruited via “Lungenfibrose e.V.” and an inpatient pulmonary rehabilitation center (“Schön Klinik Berchtesgadener Land”). Lungenfibrose e.V. is a recognized and well-established patient advocacy organization in Germany. The association aims to improve co-operation with university pneumology clinics, specialist lung clinics and lung specialists for affected patients and the promotion of public health care. Data were collected both through an online survey and paper-pencil questionnaires. All participants had the opportunity to participate either online or via paper-pencil questionnaires. The paper-pencil questionnaires were laid out at patient organization meetings in pre-paid envelopes, and the link for the online version was provided through the patient organization’s mailing lists. Separately, patients of the clinic also had the option to participate either online or via paper-pencil. The anonymously completed paper-pencil questionnaires were collected by the clinic and sent to the authors of the study. This process enabled participants to take part anonymously and free of charge. No financial benefit was offered, participation was voluntary, all patients gave written informed consent. The study was conducted in accordance with the Declaration of Helsinki and approved by the local ethics committee of PHB (Process-number: LuFi-PHB-1).

### Measures

Demographics and further ILD-related variables were collected including ILD-diagnosis, age, gender, course of disease (range: ‘slow-progression’ to ‘very-rapid-progression’).

#### Preliminary IAQ-itempool

Disease-related fears were measured by the 29 item IAQ-itempool. The items were rated on a five-point Likert-scale (0 = ‘never’ to 4 = ‘always’).

#### Further questionnaires

To assess health-related quality of life (HRQoL), the King’s Brief Interstitial Lung Disease was used (K-BILD^12,20,11^). It is divided into three domains (‘Breathlessness-and-Activity’, ‘Chest-Symptoms’, ‘Psychological-Impact’). The values are converted into a scale from 0-100, with 100 reflecting the best HRQoL. The questionnaire shows good reliability and validity.^12^ The Generalized Anxiety Disorder scale (GAD-7^21^) measures the severity of general anxiety, with higher scores representing greater anxiety.^22^ It shows good psychometric properties.^21,23^ Discriminant validity was examined using the Locus-of-Control about Disease and Health Questionnaire (German: KKG^24^). It measures a person’s beliefs about the course and manageability of their physical ailments. We used the internal Locus-of-Control scale (7 items), which shows good psychometric properties. Higher scores indicate a greater locus of control.^24^

### Analyses

SPSS, version 28 was used. For IAQ-calculation, 0.30% of missing values were tolerated. Indicating that data were missing completely at random, we used the multiple imputation strategy Expectation-Maximization.^25^ Item analyses (skewness, kurtosis) were conducted using the entire initial IAQ-itempool. For item reduction we used an exploratory test construction strategy, implemented through several successive exploratory factor analyses (principal component analyses, promax rotation).^26^ Items with the lowest factor loadings, content similarities and ambiguity were excluded. Factors were extracted using the scree plot and an additional parallel analysis. After selecting the final items, we did a principal axis analysis (promax rotation) to verify if the results are method invariant. The resulting factor solution was tested with structural equation modeling (SEM) in AMOS, serving as a confirmatory method. Afterwards, we assessed the reliability of the subscales (Cronbach’s α). Furthermore, corrected item-total correlations were evaluated (for details see Supplement S1: Construction of the final Questionnaire through Item-, Reliability- and Factor Analyses, referred to as ‘Main Analyses’).

Moreover, construct validity was assessed (Convergent: K-BILD, GAD-7; Discriminant: KKG, for details see Supplement S1: Assessing Construct Validity for the Final Questionnaire). Furthermore, we examined preliminary normative data. In addition, we used hierarchical regression analyses to examine the impact of the various disease-related fears on different aspects of HRQoL. Four analyses were conducted using the K-BILD as an outcome (for each K-BILD domain as well as the total score). Details are described in the results section. Item- and factor analyses on the IAQ as well as normative data were carried out with *N* = 166 patients (main analyses). To maintain a conservative approach in our analyses, we did not perform multiple imputation on the mostly very short validation questionnaires (e.g., GAD-7, KKG-internal: 7 Items, K-BILD subscales: 3 to 7 items). Whenever a person showed a missing value in any of the validation questionnaires, all validation questionnaires for that individual were deleted, although the ‘main data’ collected from the participants remained intact. The exclusion of cases with missing data from the further analysis was based on a very conservative approach: We wanted to exclude the possibility that imputation of items in these short validation questionnaires might disproportionately influence the results, which could lead to greater bias and reduce the power of the analyses of convergent and discriminant validity. Therefore, we decided not to use imputation strategies but rather to exclude patients with further incomplete data (for details see Supplement S1: Rationale for Imputation/Exclusion of Validation Questionnaires with Missing Data). The additional validation questionnaires were completely answered only by a subgroup (*N* = 126). There were no differences between the groups in terms of age, gender, and overall burden.

## Results

### Participants

In total, 206 individuals agreed to participate in the study. Inclusion criteria were a diagnosis of interstitial lung disease (ILD), voluntary participation, and written informed consent. After screening, 40 participants were excluded, mostly for unsuitable diagnoses (e.g., comorbid COPD), incomplete diagnostic information (those unable to specify their ILD type were excluded), or failure to complete the item pool (over 30% missing responses). Finally, we included 166 patients in our analyses. The sample consisted of 96 men (57.8%) and 67 women (40.4%), 3 people did not specify their gender (1.8%). Age ranged from 27 to 86 years with a mean age of 67.05 years (*SD* = 11.45). The majority of patients were diagnosed with IPF (94, 56.6%), further diagnoses are listed in Table 1. Overall average HRQoL was 52.42 (*SD* = 17.33, Table 1).

**Table 1.** Sample characteristics.

Characteristics	Data
Diagnosis, no. (%)	
Idiopathic pulmonary fibrosis	94 (56.6)
Hypersensitivity pneumonitis	27 (16.3)
Idiopathic nonspecific interstitial pneumonia (iNSIP)	21 (12.7)
Systemic disease associated ILD	12 (7.2)
Sarcoidosis	5 (3)
Other interstitial lung diseases	7 (4.2)
Age, mean in years (SD)	
Overall	67.05 (11.45)
Female	65.31 (11.34)
Male	68.55 (11.12)
No response	1 (0.6)
Sex, no. (%)	
Female	67 (40.4)
Male	96 (57.8)
No response	3 (1.8)
BMI, mean (SD)	
Overall	27.17 (4.98)
No response	4 (2.4)
Partner status, no. (%)	
Married or partner	140 (84.34)
Divorced, widowed, unmarried	26 (15.66)
Course of disease, no. (%)	
Slow progression	62 (37.3)
Progressive	33 (19.9)
Stable disease	32 (19.3)
Rapid progression	12 (7.2)
Very rapid progression	3 (1.8)
I don’t know/ no response	24 (14.46)
Heath related quality of life, mean (SD)	
K-BILD total	52.42 (17.33)
K-BILD breathlessness-and-activity	38.62 (24.01)
K-BILD psychological-impact	53.53 (17.75)
K-BILD chest-symptoms	60.06 (21.88)

*Note*: No.: number. SD: standard deviation. Data are presented as number or percentage unless otherwise indicated.

#### Factor structure and reliability

After item reduction was completed, the final IAQ was created with a total of 18 items (for details see Supplement S3). These were distributed among five scales: Fear-of-Dependence-and-Progression (FP), Fear-of-Social-Exclusion-and-Isolation (FSE), Fear-of-Physical-Activity (FPA), Fear-of-Dyspnea (FD), and Sleep-related-Complaints (SRC; for details see Supplement S4). The cumulated explained variance was 72.18%. Subsequent analyses showed that the final factor structure was found to be method invariant: The principal axis analysis yielded similar results. The resulting five factor solution of the ILD-Anxiety-Questionnaire (IAQ) mostly replicated the five factor solution found in COPD-patients (COPD-Anxiety-Questionnaire, CAF-R^14^). For a detailed examination of the minor differences in the content emphasis of the scales, see the discussion.

**Figure 1.**
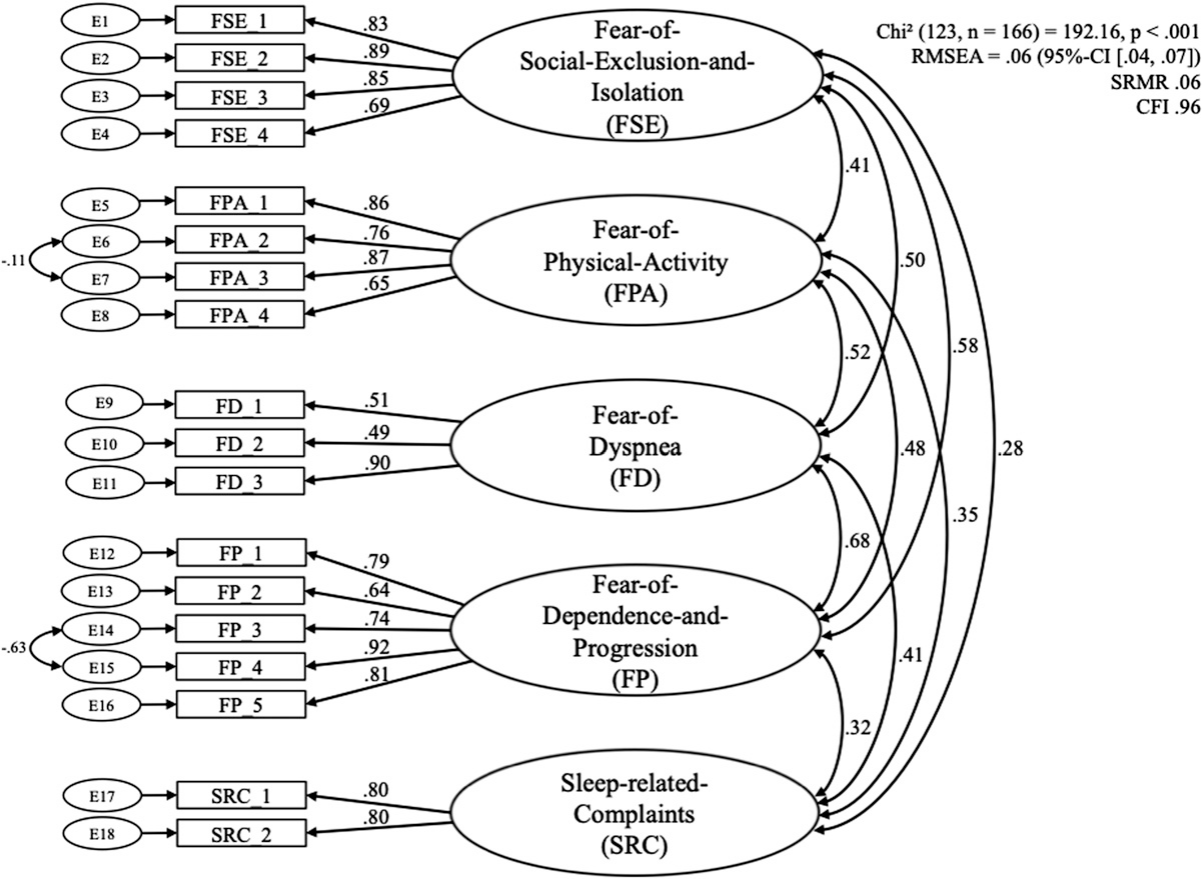
Structural equation model. Standardized regression weights are depicted between items and subscales as well as correlations among the subscales; statistics is reported as well as fit indices RMSEA (95% confidence interval), SRMR, and CFI. RMSEA: root mean square error of approximation; SRMR: standardized root mean residual; CFI: comparative fit index.

Moreover, the replicated factor solution was tested confirmatory with a SEM in AMOS (Figure 1). The fit indices indicated a good model fit (root mean square error of approximation, RMSEA = 0.06; 95% confidence interval [0.04, 0.07]), standardized root mean residual, SRMR = 0.06; and the comparative fit index (CFI, 0.96) indicated a good model fit (Cutoff-criteria: SRMR = 0.08, RMSEA = 0.06 (absolute fit indices, the lower the better), CFI = 0.95 (incremental fit index, the higher the better).^27^ The χ^2^ test statistic yielded significance at χ^2^ (123, *n* = 166) = 192.16, *p* < .001).

Internal consistency and reliability were assessed using Cronbach’s *α*. All five subscales showed acceptable to high reliability (*α =* 0.68-0.89, Table 2). Cronbach’s *α* over all scales was 0.90. The corrected item-total-correlation ranged from *r*_itc_ = .61-0.80 for each scale.

**Table 2.** Validity and reliability of the IAQ.

Items	IAQ				
	FP	FSE	FPA	FD	SRC
Convergent, disease-related					
K-BILD breathlessness-activity	−.53***	−.44***	−.74***	−.45***	−.35***
K-BILD psychological-impact	−.70***	−.55***	−.46***	−.50***	−.33***
K-BILD chest-symptoms	−.61***	−.46***	−.48***	−.49***	−.41***
K-BILD total	−.71***	−.56***	−.63***	−.54***	−.41***
Convergent, general					
GAD-7	.60***	.52***	.30**	.50***	.31**
Discriminant					
KKG internality	−.19*	−.18*	−.17	−.07	.02
Cronbach’s α	.88	.89	.86	.68	.78

Notes: IAQ: ILD-Anxiety-Questionnaire; GAD-7: Generalized Anxiety Disorder 7; KKG: Locus of Control About Disease and Health Questionnaire; K-BILD: King’s Brief Interstitial Lung Disease. FP = Fear-of-Dependence-and-Progression; FSE = Fear-of-Social-Exclusion-and-Isolation; FPA = Fear-of-Physical-Activity; FD = Fear-of-Dyspnea; SRC = Sleep-related-Complaints. *** = *p* < .001; ** = *p* < .01; * = *p* < .05.

### Construct validity

All scales of the final IAQ showed the expected correlations with convergent and discriminant constructs (*r*=.02 lowest discriminant, 0.74 highest convergent). More specifically, the various ILD-related fears showed moderate to high correlations with HRQoL. As hypothesized, general anxiety also correlated with disease-related anxiety, but significantly less than disease-related concepts. To test discriminant validity, we examined the association between disease-related anxiety and patients’ Locus-of-Control. As hypothesized, lower, nonsignificant correlations emerged (*r* = 0.02-0.19, Table 2).

### Regression

The contribution of ILD-related fears to HRQoL was analyzed with stepwise multiple linear regressions. The analyses were performed for each K-BILD domain and the total score as outcome. In three consecutive hierarchical steps, covariates (age, gender), course of disease and general anxiety (GAD-7) were entered cumulatively. The IAQ-sum-score was added in the final step. Moreover, we also examined the differential impact of the subscales on HRQoL. Therefore, the stepwise multiple linear regression was additionally performed with all IAQ-scales together in the final step.

#### K-BILD-total-score

Results of the stepwise regression show, that the IAQ-sum-score predicted the K-BILD-total-score (*ß* = −3.96, *p* < .001) over and above demographics, course of disease and general anxiety. Moreover, in the second regression, the addition of the IAQ-scales improved the explained variance by 0.19 up to 0.65 (adjusted *R*^2^). Both Fear-of-Physical-Activity and Fear-of-Dependence-and-Progression proved as predictors for HRQoL (*ß* = −0.30, *p* < .001 and *ß* = −0.30, *p* < .01) over and above GAD-7 (*ß* = −0.23, *p* < .01), course of disease and demographics (Table 3).

**Table 3.** Regression Analysis. Associations of ILD-related fears (IAQ) with health-related quality of life (K-BILD) cross-domain.

Model	B	SE	*ß*	*p*-value	*R* ^*2*^
(1) Covariates					−0.00
Age	0.04	0.09	0.03	n.s	
Gender	−1.92	2.01	−0.05	n.s	
(2) ILD-status variables					0.24
Course of disease	−1.43	0.77	−0.12	n.s	
(3) Psychological symptomatology					0.46
GAD-7	−0.94	0.29	−0.23	<.01	
(4) ILD-related fears					0.65
Fear-of-Physical-Activity	−1.32	0.30	−0.30	<.001	
Fear-of-Dependence/Progression	−0.96	0.30	−0.28	<.01	
Fear-of-Dyspnea	−0.34	0.45	−0.05	n.s	
Fear-of-Social-Exclusion/Isolation	−0.30	0.33	−0.07	n.s	
Sleep-related-Complaints	−0.66	0.61	−0.06	n.s	

*Note*: IAQ: ILD-Anxiety-Questionnaire; GAD-7: Generalized Anxiety Disorder 7; K-BILD: King’s Brief Interstitial Lung Disease. Models’ explained variances are reported as adjusted *R*^2^. Significance of increase in models explained variance (*p* < .01). n.s.: not significant.

#### K-BILD-subscales

Breathlessness-and-Activity: The IAQ-sum-score served as a predictor (*ß* = −5.54, *p* < .001) over and above demographics, course of disease and general anxiety. Regarding the IAQ-subscales as predictors, results showed that Fear-of-Physical-Activity in particular was important for Breathlessness-and-Activity (*ß* = −0.58, *p* < .001).

Psychological-Impact: Again, the IAQ-sum-score served as a predictor (*ß* = −3.09, *p* < 0.001) over and above the other factors. Regarding the IAQ-subscales as predictors, results showed that Fear-of-Dependence-and-Progression in particular was important for Psychological-Impact (*ß* = −0.35, *p* < .001).

Chest-Symptoms: The IAQ-sum-score served as a predictor (*ß* = −4.61, *p* < .001) over and above the other factors. Regarding the IAQ-subscales as predictors, results showed that Fear-of-Dependence-and-Progression (*ß* = −0.31, *p* < .01) and Sleep-related-Complaints (*ß* = −0.15, *p* < .05) showed additional incremental variance across the other factors.

### Normative data

Percentiles were calculated to obtain normative data for the IAQ-subscales. Due to significant gender differences in Fear-of-Dependence-and-Progression (*p* > .05), they are presented based on gender. For the other scales, we present common norms for both genders (Table 4).

**Table 4.** IAQ norms presented in percentiles, total scores.

Percentiles	IAQ				
	FP Female/male	FSE	FPA	FD	SRC
10	5/3	1	2	1	0
20	6/5	2	4	2	0
30	10/7	4	5	3	1
40	11/9	5	6	4	1
50	13/10	6	8	5	2
60	14/11	8	9	5	2
70	15/13	9	10	6	3
80	16/15	10	12	7	4
90	18/18	11	13	8	5
95	20/20	13	14	9	6
99	./.	15	16	12	7

*Note*: IAQ: ILD-Anxiety-Questionnaire. FP = Fear-of-Dependence-and-Progression; FSE = Fear-of-Social-Exclusion-and-isolation; FPA = Fear-of-Physical-Activity; FD = Fear-of-Dyspnea; SRC = Sleep-related-Complaints. Total scores ranged between 0 and 20 (FP), 0 and 16 (FSE, FPA), 0 and 12 (FD) and 0 and 8 (SRW). The results were rounded up to two decimal places.

## Discussion

Psychological comorbidities are common in ILD. Although we know from studies in other chronic lung diseases (i.e. COPD^14–16^) that disease-related fears play an important role for QoL, anxiety in ILD is typically only evaluated as a general psychological condition. Moreover, there is no specific measurement tool to systematically assess several ILD-related fears. In this study, we (1) identified several important ILD-related fears, (2) developed a corresponding measurement instrument to assess these fears (ILD-Anxiety-Questionnaire, IAQ) and (3) examined their importance for HRQoL.

The newly developed questionnaire consists of 18 items. The IAQ proved to be a short, valid instrument with good psychometric properties. The disease-related fears that were found to be relevant can be classified into five different dimensions: Fear-of-Dependence-and-Progression, Fear-of-Social-Exclusion-and-Isolation, Fear-of-Physical-Activity, Fear-of-Dyspnea and Sleep-related-Complaints represented in the different IAQ-scales. Moreover, in our study, we could show, that the ILD-related fears served as predictors of HRQoL, even after we statistically controlled for demographics, course of disease and general anxiety. These results suggest that ILD-related fears play an important role in the ILD-patient’s subjective experience that exceed the effects of general unspecific forms of anxiety.

As expected, these newly elaborated ILD-related fears show some similarities to the disease-related fears known to be relevant in other chronic lung diseases. But there are also some specific aspects due to the unique characteristics of ILD. Most similar to COPD-related fears are certainly Fear-of-Dyspnea and Fear-of-Physical-Activity. Fear-of-Dyspnea describes increased worries about the ultimate consequences of dyspnea (i.e. ‘Fear-of-suffocation’) that evoke whenever shortness of breath occurs. Fear-of-Physical-Activity refers to the fear of exhausting, or breath accelerating activities. Fear-of-Dyspnea and even the anticipation of dyspnea itself can strongly influence patients’ emotions and behavior, with patients often restricting their physical activities to avoid dyspnea.^28^ From studies with COPD-patients we know that especially Fear-of-Physical-Activity and Fear-of-Dyspnea are associated with worse functional exercise capacity and physical HRQoL.^29,30,14,16^ In particular, lower physical activity is a predictor of poorer health in status in COPD,^31^ highlighting the importance of addressing Fear-of-Physical-Activity. Consistently, in our study Fear-of-Dyspnea and Fear-of-Physical-Activity predicted Breathlessness-and-Activity, even after we statistically controlled for demographics, course of disease and general anxiety.

A very important concept in chronic physical illnesses in general is Fear-of-Dependence-and-Progression. Fear-of-Dependence-and-Progression is also common in COPD and many studies have shown its impact on peoples’ QoL.^14^ Interestingly, it is also one of the most frequent distress symptoms of cancer-patients.^32^ Currently, it has been described for the first time in ILD. A recent study shows, that it plays a crucial role for QoL and, moreover, mediates the association between illness perceptions and QoL.^33^ In our sample, Fear-of-Dependence-and-Progression was the most pronounced fear (it includes the fear of needing extensive care and becoming a burden to others). From studies on COPD-patients we learned that this fear is often associated with End-of-Life-Fears and diminished QoL.^13^ Consistently, Fear-of-Dependence-and-Progression in our study was strongly correlated with K-BILD Psychological-Impact. This demonstrates that in ILD Fear-of-Dependence-and-Progression reflects the psychological distress that arise from progression and its’ anticipation and also the connection to respiratory symptoms.

Fear-of-Social-Exclusion-and-Isolation can be described as the feeling of being perceived only as “the sick person” and being excluded from daily life. Some studies are already highlighting the major negative impact of loneliness for disease-related outcomes in chronic lung diseases.^34^ Studies in COPD already show the importance of Fear-of-Social-Exclusion-and-Isolation for QoL.^13^ However, patients with rare diseases such as ILD, often have to deal with a number of problems, that make these fears particularly relevant: Feeling “invisible” to the healthcare systems, the paucity of experts, the lack of appropriate treatments, and social exclusion faced by patients and families (cf.^35^). Hence, we considered these aspects when developing the scale. Consistently, in our study Fear-of-Social-Exclusion-and-Isolation strongly correlates with K-BILD Psychological-Impact.

The IAQ was developed to assess disease-related anxieties that have demonstrated relevance in other chronic lung diseases. Importantly, the factor solution found in COPD was replicated within the IAQ.^14^ The validity was largely supported by correlations with established questionnaires. In absence of directly validated instruments measuring various ILD-related anxieties, we utilized related constructs to demonstrate convergent validity. We did not expect very high correlations, since the constructs used were either disease-related but not capturing anxieties (i.e. K-BILD) or capturing general anxiety, but not disease-related (GAD-7). In line with our expectations (cf.^19,13,14^), the IAQ-scales each showed the highest correlations to disease-related concepts (K-BILD-total-score) and moderate, but significant correlations to general anxiety. To assess discriminant validity, we used the KKG, which measures control beliefs of illness and health. A high internal-control-belief score indicates that a person believes they can control illness-related events through their own actions. We chose this scale because it describes how people deal thematically with their illness without directly measuring psychopathology. In line with our expectations, there were weak to no correlations with all IAQ-scales (*r* = 0.19 to 0.06). In summary, the validity of the IAQ scales was well established.

### Limitations

First, due to the cross-sectional design, causal relationships cannot be established with certainty. Future investigations should explore the influence of disease-related anxieties over time. Second, the online data collection through a patient advocacy organization may have led to a selective sample. To gain an insight into the comparability of our sample, we compared the total burden of our ILD-patients with that of other large studies. The results are comparable.^36^ Third, patients self-assessed the course of their disease using our provided categories, which may have led to inaccuracies. To mitigate this potential bias, we incorporated the “unknown status” option. Individuals opting for this category were purposefully excluded from the regression analyses to uphold a conservative approach in addressing this concern. Overall, we conclude that our study was conducted on a sufficiently large and valid sample. Given the broad variance of the methods employed, robust and credible initial insights into disease-related anxieties in ILD can be expected.

In this study, we chose not to perform multiple imputation on the mostly very short validation questionnaires (i.e. GAD: 7 items). We wanted to ensure that the imputation of items in these short validation questionnaires does not disproportionately affect the results and diminish analytical integrity (for details, see Supplement S1). This conservative approach could potentially limit the generalizability of our findings. However, no significant differences in age, gender, or overall burden were found between groups, mitigating concerns about generalizability. These considerations should be noted when interpreting the results.

## Conclusion

The ILD-Anxiety-Questionnaire represents the first instrument to assess various disease-related fears in ILD-patients. In our study, we were able to demonstrate that ILD-related fears had an impact on HRQoL. These findings are consistent with those in COPD-patients (cf.^16^) and suggest that in ILD, disease-related fears could play a significant role in patients’ coping with the disease, surpassing the effects of general unspecific forms of anxiety. Therefore, the questionnaire could contribute to a deeper understanding of the psychological impact of ILD, leading to more comprehensive research on this aspect of the disease. Interestingly, the disease-related fears varied in their impact on different aspects of HQoL. This emphasizes the importance of differentiating between various disease-related fears. In particular, the IAQ could help healthcare providers better understand the psychological aspects of ILD, leading to more holistic and patient-centered care. Moreover, identifying specific anxieties could lead to tailored interventions and support services to address the unique needs of ILD-patients. By addressing disease-related anxieties, patients may experience improved QoL, which could also result in better adherence to treatment plans. Furthermore, detecting anxieties early through this questionnaire may enable healthcare providers to intervene proactively, potentially preventing the worsening of ILD and related complications.

Future studies should explore potential causal links between disease-related fears, disease progression, and HRQoL through longitudinal research. Additionally, validation studies should be conducted to assess the questionnaire’s reliability and validity across diverse ILD populations, leading to further refinement. Using the questionnaire in longitudinal studies to track changes in disease-specific anxieties over time and their relationship to ILD progression is also recommended. In conclusion, the development of the IAQ represents a significant step forward, opening new avenues of research to enhance the lives of those facing this challenging condition.

## Supplemental Material

Supplemental Material - Development and initial validation of the ILD-anxiety-questionnaire (IAQ): A new instrument for assessing disease specific fears in interstitial lung disease Supplemental Material for Development and initial validation of the ILD-anxiety-questionnaire (IAQ): A new instrument for assessing disease specific fears in interstitial lung disease by Nikola M. Stenzel, Nina Piel, Klaus Kenn and Michael Kreuter in Chronic Respiratory Disease.
