# Supplementary material for: Development and initial validation of the ILD-Anxiety-Questionnaire (IAQ): A new instrument for assessing disease specific fears in interstitial lung disease
Source: Chron Respir Dis. 2024 Aug 26;21:14799731241274785. doi: 10.1177/14799731241274785 (PMC11364163; doi:10.1177/14799731241274785)
Supplement: Supplemental Material - Development and initial validation of the ILD-anxiety-questionnaire (IAQ): A new instrument for assessing disease specific fears in interstitial lung disease [file sj-pdf-1-crd-10.1177_14799731241274785.pdf]

Supplement 1: Detailed description of the methodology used to construct the ILD Anxiety Questionnaire (IAQ) and to calculate the construct validity of the final questionnaire

Construction of the initial IAQ Item Pool (to be tested and reduced through item- and factor analyses). The development of the ILD Anxiety Questionnaire (IAQ) was based on the COPD Anxiety Questionnaire (CAF). The CAF (and it`s revised and shortened version, the CAF-R) are self-assessment tools designed to evaluate disease-related fears in COPD patients. The CAF (long-version) comprises 28 items in its main item pool, rated on a five-point Likert scale (0 = 'never' to 4 = 'always').

In the first step, we used the CAF items as the basis for the initial IAQ item pool. We engaged two ILD experts actively involved in clinical and scientific practice (i.e., INSIGHTS-IPF registry) who lead large clinical facilities dedicated to treating ILD patients. Both experts have over 15 years of extensive experience in clinical settings and research within this field. Experts did not provide informed consent since no personal information was disclosed. The experts suggested eliminating the CAF item "I feel ashamed when I have to cough in public." because it was specifically designed to depict feelings of shame and guilt in COPD patients. They recommended incorporating the remaining CAF items into the IAQ item pool.
Next, the experts collaborated with patient representatives, presenting them with the initial item pool to identify any potential omissions. The patient representatives were randomly selected from a sample of inpatients in these clinical facilities who had agreed, upon admission, to be approached in research projects. At this stage, the patient representatives did not directly complete or rate any of the items. Instead, based on their own experience, they highlighted additional disease-specific aspects (such as the disease's limited public awareness) that should be included in the IAQ item pool.

Suggestions from patients and experts were discussed within the team, leading to the formulation of two additional items ("I feel that those around me [e.g., family, acquaintances] do not understand me well because interstitial lung diseases are not well-known" and "The fact that interstitial lung diseases are so unknown results in little understanding from my environment"). Our team then refined these items, eliminating redundancies and addressing inconsistencies. Following a comprehensive review and refinement process led by the experts, the initial item pool was finalized to include 29 items (ILD Anxiety Questionnaire Item Pool).

Construction of the final Questionnaire through Item-, Reliability- and Factor Analyses (referred to as 'Main Analyses'). Item analyses (skewness, kurtosis) were conducted using the entire initial IAQ item pool. For item reduction, we used an exploratory test construction strategy, implemented through several successive exploratory factor analyses (principal component analyses, promax rotation). Items with the lowest factor loadings, content similarities and ambiguities were excluded. Factors were extracted using the scree plot and an additional parallel analysis. After selecting the final items, we conducted a principal axis analysis (promax rotation) to verify the method's invariance. The resulting factor solution was tested with structural equation modeling (SEM) in AMOS, serving as a confirmatory method. According to Beauducel and Wittmann[1], the confirmatory factor analysis (CFA) utilized the following fit indices and criteria: Root Mean Squared Error of Approximation (RMSEA, cut-off ≤ 0.08), Standardized Root Mean Square Residual (SRMR; cut-off ≤ 0.11), Comparative Fit Index (CFI; cut-off ≥ 0.95), and the Chi-Square Test. Additionally, the analysis included a check for multivariate normality. We then assessed the reliability of the subscales (Cronbach's α) and evaluated corrected item-total correlations. Coefficients of discrimination below 0.3 are considered low, between 0.3 and 0.5 are moderate, and above 0.5 are high.

Assessing Construct Validity for the Final Questionnaire. In order to classify the final ILD Anxiety Questionnaire (IAQ) within a system of construct-related and non-construct-related measurement instruments, we evaluated its construct validity. Convergent validity (the ability of a questionnaire to measure conceptually similar constructs as other already established instruments) was examined using the Generalized Anxiety Disorder scale (GAD-7), which assesses general anxiety. Additionally, we used the King's Brief Interstitial Lung Disease (K-BILD), which measures disease specific quality of life across three different scales. Discriminant validity (ensuring that measurements of different constructs differ from each other) was evaluated using the internal Locus-of-Control scale from the Locus-of-Control about Disease and Health Questionnaire (German: KKG). Pearson product-moment correlations were used to analyze the relationships between IAQ scores and the measures of convergent and discriminant constructs.

Rationale for Imputation/ Exclusion of Validation Questionnaires with Missing Data. For the IAQ calculation, 0.30% of missing values were tolerated. Assuming that data were missing completely at random, we employed the Expectation-Maximization (EM) multiple imputation strategy for the IAQ items. This technique estimates the mean, variance, and covariance from individuals whose data is complete. Using these estimates, maximum likelihood procedures were applied to derive regression equations that relate each variable to the others. Finally, the missing values were estimated. This iterative process, performed with SPSS by default, engaged up to 25 iterations until the estimates changed only negligibly[2]. We chose this strategy due to its solid statistical and theoretical foundation, as well as its efficiency and accuracy compared to simple imputation methods.

However, for the additional questionnaires (GAD-7, KKG, K-BILD), we chose a different path primarily due to their short length and their use for construct validation purposes. The GAD-7and the KKG (internal scale) both consist of 7 items, and the K-BILD-subscales consists of 3 to 7 items per scale (that is rather short, compared to the initial IAQ item pool of 29 items). We wanted to exclude the possibility that imputation of items in these short questionnaires might disproportionately influence the results, which could lead to greater bias and reduce the power of the analyses of convergent and discriminant validity. In particular, because we wanted to compare the questionnaire with other constructs and established measurement instruments, we depended on them measuring as accurately as possible what they were intended to measure. Therefore, we decided not to use imputation strategies but rather to exclude patients with further incomplete data.

The exclusion of cases with missing data from the further analysis was based on a very conservative approach. By removing all cases with missing information, the analysis was limited to the cases with complete answers, thereby maintaining the robustness of the results. Both groups did not differ in terms of age, gender, and total burden. This method was chosen to ensure the integrity of the dataset by avoiding potential biases and inaccuracies arising from incomplete data.

References cited in the supplemental material

1. Beauducel A, Wittmann WW. Simulation Study on Fit Indexes in CFA Based on Data With Slightly Distorted Simple Structure. *Struct Equ Model Multidiscip J* 2005; 12: 41–75.
2. Schafer JL, Olsen MK. Multiple Imputation for Multivariate Missing-Data Problems: A Data Analyst’s Perspective. *Multivar Behav Res* 1998; 33: 545–571.

Table S2: *ILD-Anxiety-Questionnaire (IAQ), Initial Item Pool*

Items original German version	Items translated English version*
Instruktion: Bitte bearbeiten Sie jede Aussage, indem Sie die Antwort (Zahl) ankreuzen, die auf	Instruction: Please mark the appropriate answer (number) for each statement.

Sie zutrifft.	
1. Ich wache nachts wegen meiner Kurzatmigkeit auf.	I wake up at night because of my shortness of breath.
2. Ich vermeide körperliche Anstrengung.	I avoid physical exertion
3. Ich glaube, dass andere nicht nachvollziehen können, wie es mir geht.	I don't think others can understand how I feel.
4. Wenn ich kurzatmiger werde, mache ich mir Sorgen, ich könnte gleich ersticken.	When I get short of breath, I worry that I might suffocate.
5. Ich stelle mir in Gedanken den Weg ganz genau vor, bevor ich losgehe.	I visualize the route in my mind before I set off.
6. Der Gedanke, von anderen abhängig zu sein, macht mir Angst.	The thought of being dependent on others scares me.
7. Ich fühle mich nur sicher, wenn ich in der Klinik, beim Arzt oder in einer anderen medizinischen Einrichtung bin.	I only feel safe when I am in the clinic, at the doctor's or in another medical facility.
8. Ich fühle mich mit meiner Krankheit alleingelassen.	I feel left alone with my disease.
9. Ich vermeide Aktivitäten, die mich zum Schwitzen bringen.	I avoid activities that make me sweat.
10. Die Tatsache, dass interstitielle Lungenerkrankungen so unbekannt sind führt dazu, dass mir mein Umfeld wenig Verständnis entgegenbringt.	The fact that interstitial lung diseases are so unknown results in little understanding from my environment.
11. Wenn ich kurzatmiger werde, mache ich mir Sorgen, eine Erkältung könnte im Anmarsch sein.	When I become short of breath, I worry that I might be catching a cold.
12. Ich fürchte, die Ärzte können mir nicht mehr helfen.	I fear that the doctors can no longer help me.
13. Ich habe Angst, dass meine Atembeschwerden noch schlimmer werden.	I am afraid that my breathing difficulties will get worse.
14. Ich schäme mich, dass ich nicht mehr so leistungsfähig bin wie früher.	I am ashamed that I am not as productive as I used to be.
15. Wenn ich kurzatmiger werde, habe ich Angst, eines Tages zu ersticken.	If I get short of breath, I'm afraid I'll suffocate one day.
16. Ich fühle mich durch die Krankheit einsam.	I feel lonely because of my disease.
17. Wenn ich kurzatmiger werde, möchte ich von einem Arzt untersucht werden.	When I start to feel short of breath, I want to be examined by a doctor.
18. Ich fühle mich mit meiner Krankheit von meinem Umfeld nicht verstanden.	I have the feeling that my environment does not understand my illness.
19. Wenn ich kurzatmiger werde, bekomme ich Angst.	When I get short of breath, I get scared.
20. Ich habe immer das Gefühl, dass andere meine Situation nicht verstehen können.	I always have the feeling that others cannot understand my situation.
21. Ich vermeide Aktivitäten, die meine Atmung beschleunigen.	I avoid activities that accelerate my breathing.
22. Meine Atemgeräusche oder mein Husten wecken mich nachts.	My breath sounds or my coughs wake me up at night.

23. Ich befürchte, dass ich durch die Krankheit vereinsame.	I fear that my disease will make me lonely.
24. Ich habe Angst, durch meine Krankheit eines Tages anderen zur Last zu fallen.	I am afraid to become, due to my illness, a burden to others one day.
25. Ich befürchte eines Tages aufgrund der Erkrankung pflegebedürftig zu werden.	I fear that one day I will be in need of care due to my illness.
26. Ich vermeide möglichst jede körperliche Bewegung.	I try to avoid almost any physical activity.
27. Ich befürchte, andere Menschen nehmen mich nur noch als Kranke/n wahr.	I fear that other people only perceive me as a sick person.
28. Wenn ich kurzatmiger werde, mache ich mir Sorgen, dass es sich zur Atemnot steigern könnte.	When I get short of breath, I worry that it could increase to breathlessness.
29. Ich habe das Gefühl, dass ich in meinem Umfeld (z.B. Familie, Bekannte) schlecht verstanden werde, weil interstitielle Lungenerkrankungen so unbekannt sind.	I feel that those around me (e.g., family, acquaintances) do not understand me well because interstitial lung diseases are not well-known.

** Note: This is an initial and preliminary translation of the original questionnaire. It has not yet been psychometrically evaluated. Validation studies for this translation are currently in preparation.*
Antwortschema: 0 = nie, 1 = selten, 2 = manchmal, 3 = oft, 4 = immer; Response scheme: Response scheme: 0 = never, 1 = rarely, 2 = sometimes, 3 = often, 4 = always

Table S3: *ILD-Anxiety-Questionnaire (IAQ), Final Version**

Items original German version	Items translated English version*
Instruktion: Bitte bearbeiten Sie jede Aussage, indem Sie die Antwort (Zahl) ankreuzen, die auf Sie zutrifft.	Instruction: Please mark the appropriate answer (number) for each statement.
1. Ich wache nachts wegen meiner Kurzatmigkeit auf.	I wake up at night because of my shortness of breath.
2. Ich vermeide körperliche Anstrengung.	I avoid physical exertion
3. Der Gedanke, von anderen abhängig zu sein, macht mir Angst.	The thought of being dependent on others scares me.
4. Ich vermeide Aktivitäten, die mich zum Schwitzen bringen.	I avoid activities that make me sweat.
5. Die Tatsache, dass interstitielle Lungenerkrankungen so unbekannt sind führt dazu, dass mir mein Umfeld wenig Verständnis entgegenbringt.	The fact that interstitial lung diseases are so unknown results in little understanding from my environment.
6. Wenn ich kurzatmiger werde, mache ich mir Sorgen, eine Erkältung könnte im Anmarsch sein.	When I become short of breath, I worry that I might be catching a cold.
7. Ich fürchte, die Ärzte können mir nicht mehr helfen.	I fear that the doctors can no longer help me.
8. Ich habe Angst, dass meine Atembeschwerden noch schlimmer werden.	I am afraid that my breathing difficulties will get worse.
9. Wenn ich kurzatmiger werde, möchte ich von einem Arzt untersucht werden.	When I start to feel short of breath, I want to be examined by a doctor.
10. Ich fühle mich mit meiner Krankheit von meinem Umfeld nicht verstanden.	I have the feeling that my environment does not understand my illness.

11. Wenn ich kurzatmiger werde, bekomme ich Angst.	When I get short of breath, I get scared.
12. Ich habe immer das Gefühl, dass andere meine Situation nicht verstehen können.	I always have the feeling that others cannot understand my situation.
13. Ich vermeide Aktivitäten, die meine Atmung beschleunigen.	I avoid activities that accelerate my breathing.
14. Meine Atemgeräusche oder mein Husten wecken mich nachts.	My breath sounds or my coughs wake me up at night.
15. Ich habe Angst, durch meine Krankheit eines Tages anderen zur Last zu fallen.	I am afraid to become, due to my illness, a burden to others one day.
16. Ich befürchte eines Tages aufgrund der Erkrankung pflegebedürftig zu werden.	I fear that one day I will be in need of care due to my illness.
17. Ich vermeide möglichst jede körperliche Bewegung.	I try to avoid almost any physical activity.
18. Ich habe das Gefühl, dass ich in meinem Umfeld (z.B. Familie, Bekannte) schlecht verstanden werde, weil interstitielle Lungenerkrankungen so unbekannt sind.	I feel that those around me (e.g., family, acquaintances) do not understand me well because interstitial lung diseases are not well-known.

** Note: This is an initial and preliminary translation of the original questionnaire. It has not yet been psychometrically evaluated. Validation studies for this translation are currently in preparation.*
Antwortschema: 0 = nie, 1 = selten, 2 = manchmal, 3 = oft, 4 = immer; Response scheme: Response scheme: 0 = never, 1 = rarely, 2 = sometimes, 3 = often, 4 = always

Table S4: *Principal Axis Factor Analysis (final factor solution)*

Factor Loadings

Items	Fear-of-Dependence-and-Progression (FP)	Fear-of-Social-Exclusion-and-Isolation (FSE)	Fear-of-Physical-Activity (FPA)	Fear-of-Dyspnea (FD)	Sleep-related-Complaints (SRC)
1. FP_1	.95				
2. FP_2	.87				
3. FP_3	.86				
4. FP_4	.70				
5. FP_5	.60				
6. FSE_1		.94			
7. FSE_2		.91			
8. FSE_3		.83			
9. FSE_4		.76			
10. FPA_1			.91		
11. FPA_2			.89		
12. FPA_3			.84		
13. FPA_4			.75		
14. FD_1				.85	
15. FD_2				.82	
16. FD_3				.53	
17. SRC_1					.91
18. SRC_2					.89

Note: Factor 1 = Fear-of-Dependence-and-Progression (FP); Factor 2 = Fear-of-Social-Exclusion-and-Isolation (FSE); Factor 3 = Fear-of-Physical-Activity (FPA); Factor 4 = Fear-of-Dyspnea (FD) and Factor 5 = Sleep-related-Complaints (SRC). (Extraction method: principal component factor analysis. Rotation method: promax with Kaiser normalization. The rotation has converged in 6 iterations.). Kaiser-Meyer-Olkin measure (.87) and Bartlett test of sphericity ($P < 0.001$) were appropriate. Factor loadings less than .3 were suppressed.
